# Continuous removal of thorium from aqueous solution using functionalized graphene oxide: study of adsorption kinetics in batch system and fixed bed column

**DOI:** 10.1038/s41598-024-65709-7

**Published:** 2024-06-27

**Authors:** Fazel Zahakifar, Fereshte Khanramaki

**Affiliations:** Nuclear Fuel Cycle Research School, Nuclear Science and Technology Research Institute, AEOI, P.O. Box: 11365-8486, Tehran, Iran

**Keywords:** Thorium, Graphene oxide, Adsorption, Kinetics, Fixed bed column, Chemical engineering, Nuclear chemistry

## Abstract

This article investigated the kinetic studies of thorium adsorption from an aqueous solution with graphene oxide functionalized with aminomethyl phosphonic acid (AMPA) as an adsorbent. First, the AMPA-GO adsorbent was characterized using TEM, XRD, and FTIR methods. Experiments were performed in two batch and continuous modes. In batch mode, adsorption kinetics were studied in different pH (1–4), temperature (298–328 K), initial concentration (50–500 mg L^−1^), and dosages (0.1–2 g L^−1^). The results showed that thorium adsorption kinetic follows pseudo-first-order kinetic model and that the adsorption reaction is endothermic. The maximum experimental adsorption capacity of thorium ions was observed 138.84 mg g^−1^ at a pH of 3, adsorbent dosage of 0.5 g L^−1^, and a temperature of 328 K. The results showed that AMPA-GO adsorbent can be used seven times with an acceptable change in adsorption capacity. In continuous conditions, the effect of feed flow rate (2–8 mL min^−1^), initial concentration (50–500 mg L^−1^), and column bed height (2–8 cm) was investigated. The continuous data was analyzed using the Thomas, Yoon-Nelson, and Bohart-Adams models. The experimental data of the column were well matched with the Thomas, and Yoon-Nelson models. The research results showed that the use of functionalized graphene oxide adsorbents has a great ability to remove thorium from aqueous solutions.

## Introduction

Present-day heavy metal cations in surface and subsurface water, and waste water from different industries, are a worry for the environment ^1^. Numerous environmental consequences are brought on by trace levels of heavy metals in water ^2–6^. Common and economical technologies are required to remove these cations from wastewaters. Researchers are concentrating their attention on radioactive metals since they are significant among heavy metals. This group of metals includes those harmful to human health, like thorium and uranium ^7,8^.

The extraction processes of thorium from mines, and ore processing units create a large volume of waste water during chemical processes every year. The relatively high concentration of thorium in waste water makes clear the necessity of their purification to preserve the environment's health ^9^. Common methods for removing heavy and radioactive metals include one or a combination of evaporation methods, chemical deposition ^10^, electrochemical treatment ^11^, ion exchange ^12,13^, solvent extraction ^14,15^, reverse osmosis ^16^, membrane processes ^17–21^, and adsorption ^22–24^. Each of these methods has advantages and disadvantages, and according to the conditions, each or a combination of them is selected. The adsorption process is used to remove heavy metals from aqueous solution. Therefore, the adsorption method is more interesting than other methods due to its economics, flexibility, and reproducibility ^25–32^.

Graphene oxide is a type of adsorbent with a very good ability to remove heavy metals from waste water. This adsorbent is the oxidized form of graphene ^33^. Graphene oxide can be functionalized with different functional groups. Functionalization of graphene oxide with functional groups improves selectivity and ion adsorption capacity ^34^. So far, functionalized graphene oxide has been used to remove various elements including lead, zinc, cadmium, strontium and mercury ^35–39^. A previous study described the ability to use graphene oxide adsorbent functionalized with aminomethyl phosphinic acid (AMPA-GO) for discontinuous thorium adsorption ^40^.

To reflect its novelty, this study investigates the possibility of thorium removal with AMPA-GO adsorbent in a fixed bed column. To the best of our knowledge, no similar prior study has been carried out using AMPA-GO adsorbent as a fixed bed column for thorium removal. In addition, no previous study on thorium adsorption kinetics with this adsorbent has been reported. This study looked at the kinetics of thorium adsorption on AMPA-GO adsorbent in batch and continuous mode. The kinetics of thorium adsorption on AMPA-GO adsorbent were studied in the batch mode for various parameters, including temperature, pH, initial concentration, and adsorbent dosage. In continuous mode, the effect of parameters of flow rates, bed height and different initial concentrations on the continuous removal of thorium with AMPA-GO adsorbent was investigated. Thomas, Yoon-Nelson, and Bohart-Adams models were used to evaluate the kinetic performance of the fixed bed column. The study's findings can be applied to designing a fixed bed column on a pilot plant that will extract thorium from waste water.

## Material and methods

### Laboratory materials

Potassium permanganate, sodium nitrate, and aminomethyl phosphinic acid were acquired from the Scharlou company. In contrast, strontium nitrate, nitric acid, graphite, sulfuric acid, sodium hydroxide, hydroiodic acid, acetic acid, hydrogen peroxide, and hydrochloric acid were obtained from the Merck company. Moreover, the solution containing thorium ions was prepared by dissolving a specific quantity of thorium nitrate in distilled water. The acidity of the initial solution was adjusted using either nitric acid or sodium hydroxide with a concentration of 0.1 mol L^−1^. All materials used were analytical grade and used without purification.

### Preparation of adsorbent

The modified Hummers' method was chosen for synthesizing graphene oxide in this study. Within this approach, 2 g of graphite powder and 2 g of sodium nitrate were placed on a magnetic stirrer with 50 mL of sulfuric acid under a temperature of less than 10 °C. Subsequently, a total of 6 g of potassium permanganate was gradually introduced to the mixture over 2 h. By removing the solution from the ice water bath, the temperature was elevated to 35 °C and the mixture was stirred at this temperature for 60 min. Following dilution, the solution was subjected to a bath at a temperature of 98 °C for 20 min, forming of Graphite oxide plates. 30% hydrogen peroxide was added to complete the oxidation. The resulting graphene oxide was subjected to washing with 5% hydrochloric acid and distilled water. Finally, the remaining solid was drying in an oven at a temperature of 60 °C ^41^.

The AMPA-GO synthesis process has been documented in earlier research ^40,42^. At first, in 400 cm^3^ of distilled water, about 0.2 g of GO was dissolved by sonication in an ultrasonic bath (Elmasonic, S 30H, Singen, Germany). To functionalize graphene oxide, 0.2 g of aminomethyl phosphinic acid (AMPA) extractant was added to this mixture. The resulting solution was then transferred to a refrigeration cycle balloon and subjected to stirring in an oil bath at 80 °C for 40 h at an appropriate speed to facilitate bonding between the agent and graphene oxide. Following separation via centrifugation, the sediment obtained was washed multiple times with distilled water and ultimately dried in an oven. Following separation via centrifugation, the sediment obtained was washed multiple times with distilled water and ultimately dried in an oven.

**Figure 1:**
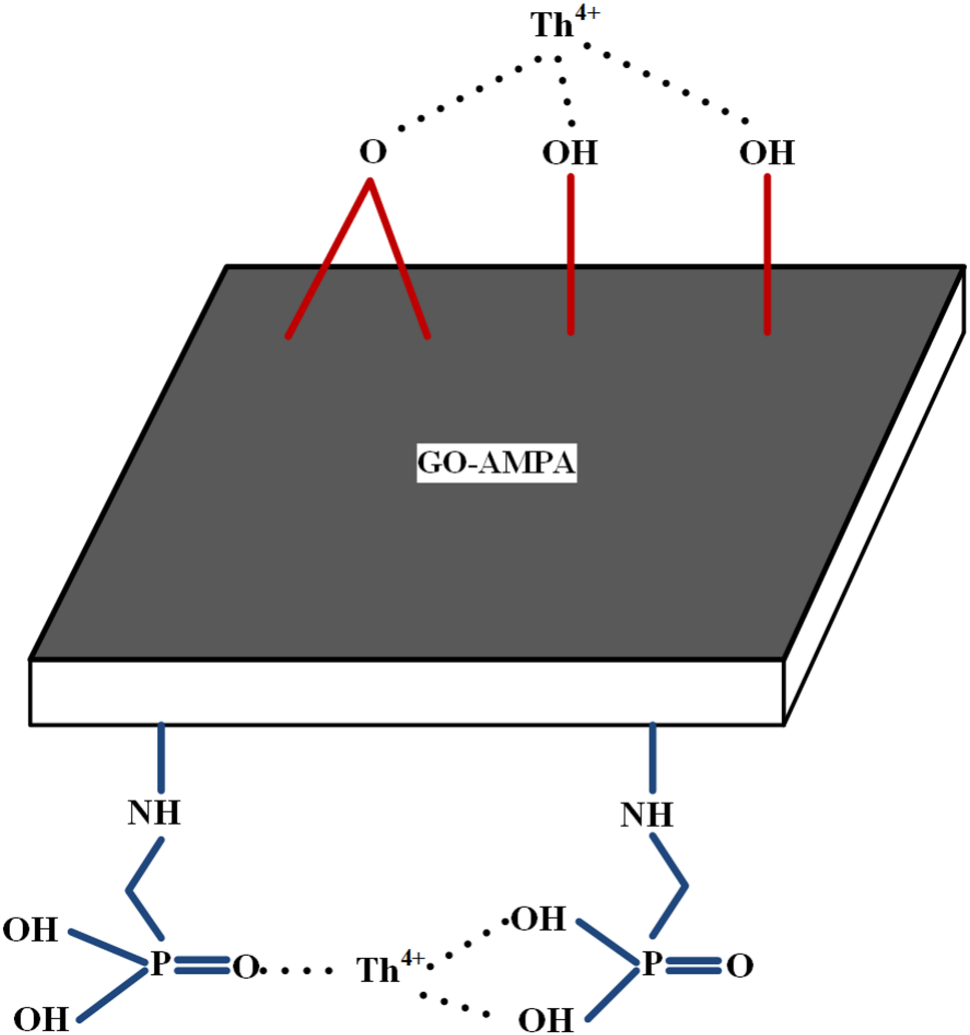
The mechanism of thorium adsorption on the graphene oxide adsorbent functionalized with aminomethyl phosphinic acid.

The adsorbent prepared through oxygen functional groups and AMPA functional group can adsorb thorium on the surface. The adsorption mechanism is shown in Fig. 1.

### Physicochemical characterization

Morphological images of the GO nanosheets and AMPA-GO hybrid were obtained using low and high-resolution transmission electron microscopy (TEM/HRTEM, JEOL, JEM-2100, Japan). The X-ray diffractometry (Rigaku Miniflex XRD, Texas, USA) and Fourier transform infrared spectroscopy (FTIR, Perkin-Elmer, SpectrumRX, USA) were also exploited to disclose the crystallographic structure and chemical bonding of the synthesized samples, respectively.

### Procedures of experiments

#### Adsorption experiments in batch system

The adsorption kinetics of thorium ions by the prepared adsorbent was investigated as a function of pH, amount of adsorbent, initial concentration of solution, and temperature. The batch adsorption experiments were carried out in flasks containing a certain amount of adsorbent and desired solution in a shaker at a speed of 200 rpm. The effective parameters of the thorium adsorption process include pH of the solution (1–4), adsorbent amount (0.1–2 g), initial metal ion concentration in the solution (50–500 mg L^−1^), and temperature (298–328 K) were studied. Thorium concentration was measured by inductively coupled plasma-atomic emission spectrometer (ICP-AES) analysis (Varian Liberty 220). The adsorption percentage of thorium and the adsorption capacity of thorium by adsorbents were calculated by the Eqs. (1) and (2), respectively:

1 $$Adsorption \left(\%\right)= \frac{{C}_{o}-{C}_{e}}{{C}_{o}} \times 100$$

2 $${q}_{e}= \frac{{C}_{o}-{C}_{e}}{\text{m}/\text{V}}$$

In these equations, C_0_ and C_e_ are the initial and equilibrium concentration of thorium solution, V is the volume of thorium solution, M is the mass of the dry adsorbent, and q_e_ is the equilibrium adsorption rate of thorium in mg g^−1^ of the adsorbent. Three experimental tests for Th adsorption were conducted, and the average outcomes were reported.

Pseudo-first-order, pseudo-second-order kinetic, and Weber- Morris models were investigated to describe thorium adsorption kinetics by functionalized graphene oxide adsorbent. The relations of pseudo-first-order Eq. (3), pseudo-second-order models Eq. (4), and Weber-Morris Eq. (5) are as follows ^43–48^:

3 $${q}_{t}={q}_{e}(1-\text{exp}\left(-{k}_{1}t\right))$$

4 $${q}_{t}=\frac{{k}_{2}{{q}_{e}}^{2}t}{1+{k}_{2}{q}_{e}t}$$

5 $${q}_{t}={k}_{i}{t}^{0.5}$$

where q_t_ and q_e_ in terms of mg g^−1^ are the values of thorium adsorption capacity at time t and equilibrium time, respectively. k_1_ in terms of min^−1^, k_2_ in terms of g mg^−1^ min^−1^, and k_i_ in terms of mg g^−1^ min^−0.5^ are the constant value of the pseudo-first-order, the pseudo-second-order model and the Weber- Morris model, respectively.

#### Adsorption experiments in continuous system (fixed bed column)

A fixed bed column with an inner diameter of 1.2 cm filled with a specific amount of adsorbent was used to perform the experiments. The feed solution containing thorium was introduced into the bed by a peristaltic pump from the bottom of the column. The concentration of thorium from the column output at certain times was determined by Inductively Coupled Plasma-Atomic Emission Spectrometer (ICP-AES) analysis.

The behavior of the column to adsorb thorium is as a graph of C_t_/C_0_ as a function of time in the desired conditions. So that C_t_ is the concentration of the ion at time t and C_0_ is the initial concentration of the metal ion.

#### Kinetic models of break through curves in fixed bed columns

The Thomas, Yoon-Nelson, and Bohart-Adams models were employed to scrutinize the data in the fixed bed column. In the Thomas model, known for its extensive utilization in investigating the efficacy of adsorption columns^38^, is derived from the mass conservation equation in a flow system. The Thomas model, it is assumed that the equilibrium of surface adsorption follows the Langmuir model, which has been shown in a previous study^39^. The mathematical expression of the Thomas model Eq. (6) is as follows:

6 $$\text{ln}\left(\frac{{C}_{0}}{{C}_{t}}-1\right)=\left(\frac{M{q}_{0}{K}_{Th}}{Q}\right)-{C}_{0}{K}_{Th}t$$

So that C_t_, C_0_, K_Th_, Q, q_0_, M and t are the ion concentration in the outlet and inlet stream, Thomas rate constant, flow rate, maximum adsorption capacity, dry adsorbent mass, and time, respectively.

Bohart-Adams model is usually used to describe the first part of the curve. The Bohart-Adams model is based on the surface reaction theory and states that the adsorption reaction is not immediate. The linear form of Bohart-Adams is given by the Eq. (7):

7 $$\text{ln}\left(\frac{{C}_{0}}{{C}_{t}}\right)=\left[\left({K}_{AB}{C}_{0}t\right)-\left(\frac{{K}_{AB}{N}_{0}Z}{{U}_{0}}\right)\right]$$

where N_0_, Z, K_AB_, and U_0_ t are the saturation concentration, column bed height, Bohart-Adams rate constant, and linear flow rate.

The Yoon-Nelson model is simple and easy. This model can be used for a one-component system. The advantage of this model is that it requires little data. The linear form of the Yoon-Nelson model is given by the Eq. (8):

8 $$\text{ln}\left(\frac{{C}_{t}}{{C}_{0}-{C}_{t}}\right)={K}_{YN}t-\tau {K}_{YN}$$

In this equation, K_YN_ and τ represent the Yoon-Nelson constant and the time required to adsorb 50% of the ion.

## Results and discussion

### Morphology and crystalline structure

**Figure 2:**
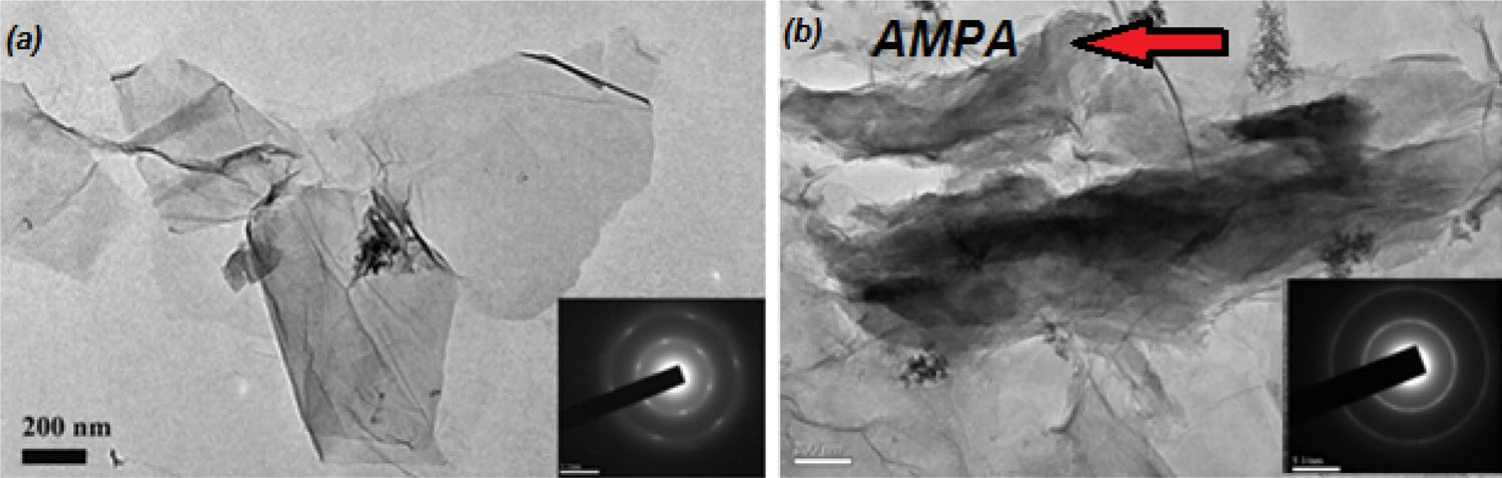
The TEM images of (**a**) graphene oxide and (**b**) graphene oxide adsorbent functionalized with aminomethyl phosphinic acid^40^.

TEM imaging was used for the microscopic study of the GO and AMPA-GO, shown in Fig. 2. The transparent regions illustrate the monolayer graphene sheets. The pictures show that the transparent graphene sheets are wavy or wrinkled. This phenomenon is especially evident at their edges, caused by the folding and turning of graphene sheets. The AMPA functional group is also indicated in the Fig. 1.

**Figure 3:**
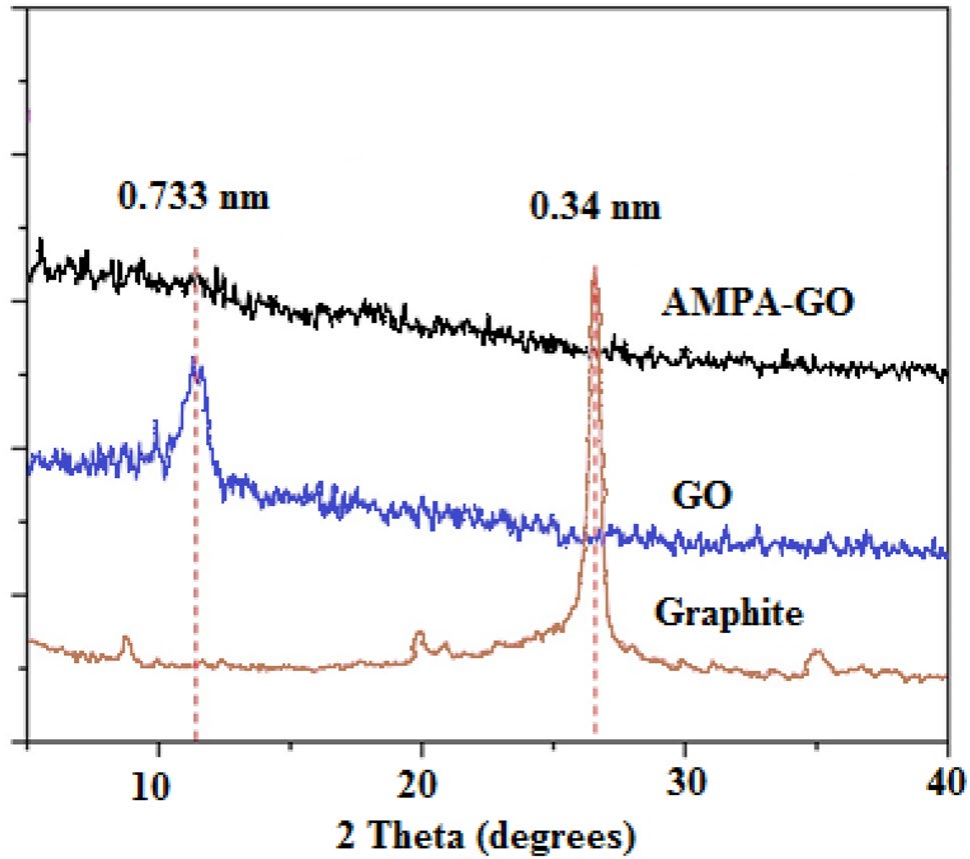
The XRD spectra of graphite, graphene oxide, and the graphene oxide adsorbent functionalized with aminomethyl phosphinic acid hybrid^40^.

Their X-ray diffraction pattern was obtained to characterize the synthesized samples' crystal structure. Figure 3 shows this pattern for the GO and AMPA-GO adsorbents.

The initial peak of graphite that occurs at 2θ = 26°, and matches to an interlayer space of 0.336 nm, was shifted to 11° for the GO corresponding to a 0.73 nm interlayer distance. From the XRD spectrum of AMPA-GO, it is clear that the material obtained has no crystalline properties, and is amorphous. The AMPA is a relatively large molecule that is polar. So, it can keep a lot of water molecules on its surface. As a result, the presence of AMPA chains between the graphite sheets causes their separation.

**Figure 4:**
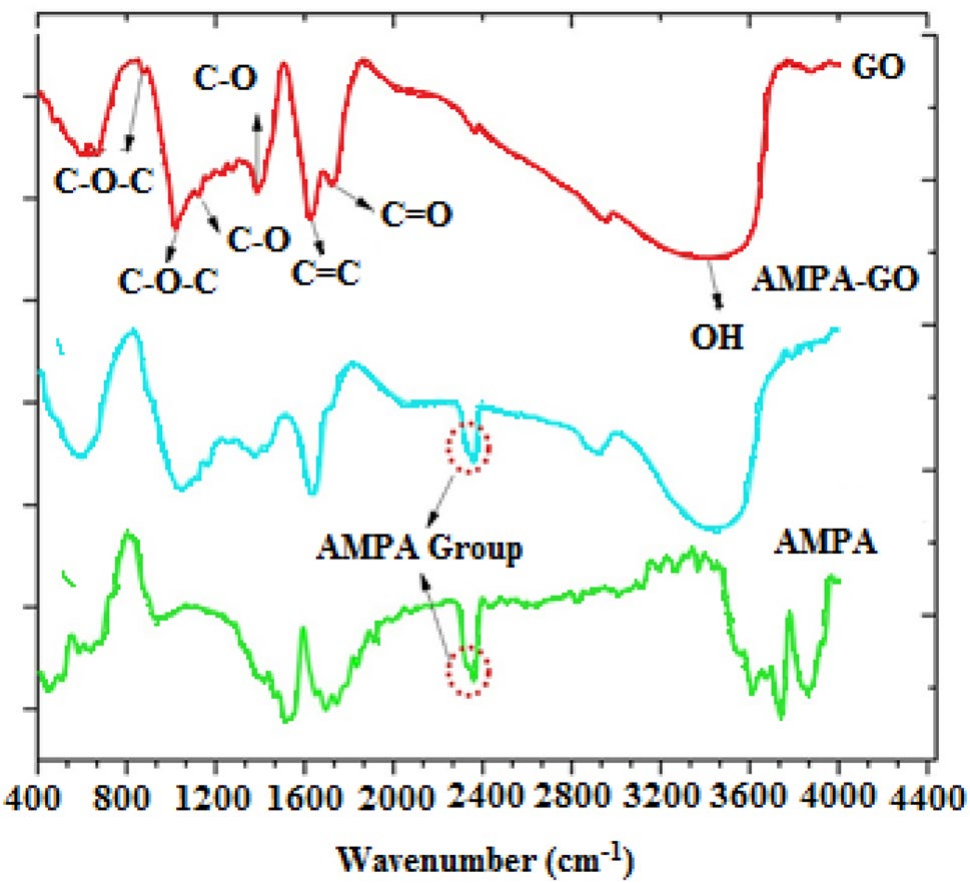
The FTIR spectra of the graphene oxide, aminomethyl phosphinic acid, and graphene oxide adsorbent functionalized with aminomethyl phosphinic acid ^40^.

FTIR spectra were performed to characterize molecular and chemical bonds and functional groups in the synthesized materials. Figure 4 shows the FTIR spectrum of GO and AMPA-GO. As can be observed, it is clear from the FTIR spectrum of GO that all major peaks associated with oxygenated functional groups are present. In the AMPA-GO spectrum, the fingerprint of the AMPA is also clearly visible at 2385 cm^−1^. Also, the carboxyl peak at 1740 cm^−1^ was completely removed in AMPA-GO due to the functionalization process. In addition, the 880 cm^−1^ and 1020 cm^−1^ peaks associated with the epoxy group have been partially removed due to functionalization. Therefore, it is well demonstrated that the AMPA functional group is attached to the GO surface. As a result, it is expected that the presence of phosphorus on GO sheets can improve strontium uptake.

### Adsorption dynamics

**Figure 5:**
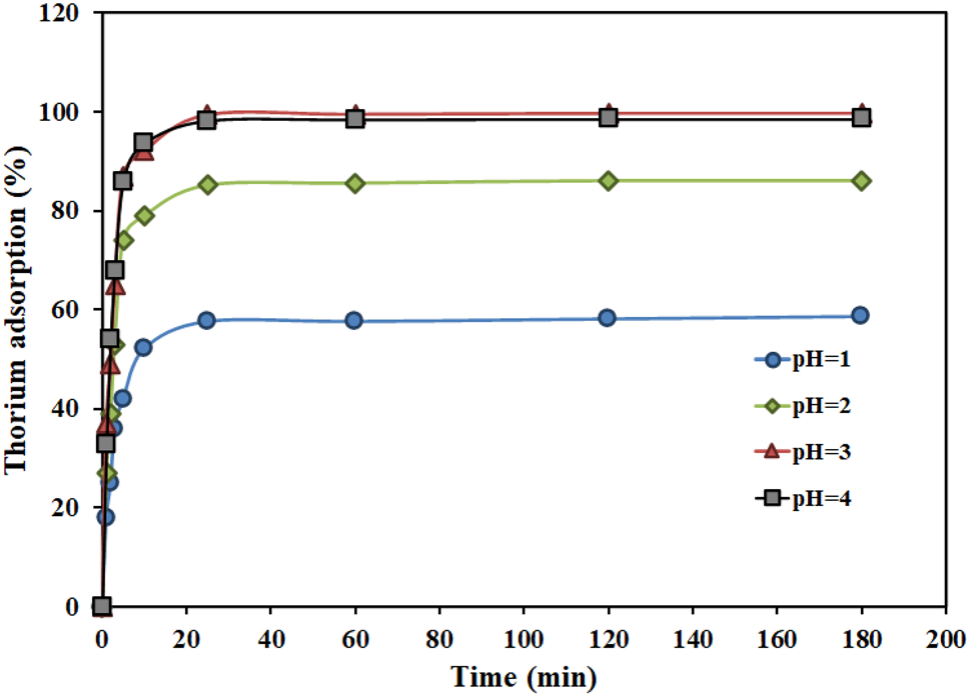
Impact of pH on the thorium adsorption onto graphene oxide adsorbent functionalized with aminomethyl phosphinic acid at various times. (Experimental conditions: Thorium concentration = 100 mg L^−1^, Adsorbent dosage = 2 g L^−1^, Time = 180 min, T = 298 K, Shaker speed = 200 rpm).

Tables 1, 2, 3 and 4 display the findings of the kinetic analysis of thorium adsorption by using AMPA-GO. The findings of Table 1 demonstrate that as the pH of the solution is raised from 1 to 3, thorium adsorption increases. At low pH, there is a competition between H^+^ and thorium ions for adsorption on the negative surface of AMPA-GO adsorbent. With the increase of pH, this competition decreases and the maximum value of adsorption occurs at pH = 3. The optimal pH for thorium adsorption with AMPA-GO adsorbent was at a pH equal to 3. (Fig. 5).

**Table 1:** Effect of pH on kinetic data thorium adsorption onto graphene oxide adsorbent functionalized with aminomethyl phosphinic acid (Experimental conditions: Thorium concentration = 100 mg L^−1^, Adsorbent dosage = 2 g L^−1^, Time = 180 min, T = 298 K, Shaker speed = 200 rpm).

Initial pH	Final pH	Adsorption (%)	q_exp_ (mg g^-1^)	Pseudo first-order			Pseudo second-order			Weber- Morris	
				k_1_	q_e (theoritical)_	R^2^	k_2_	q_e (theoritical)_	R^2^	k_i_	R^2^
1	1.06	58.50	29.25	− 0.23	36.67	98.59	0.015	30.26	99.21	1.70	57.97
2	2.17	86.10	43.05	− 0.29	41.89	98.29	0.009	44.43	97.67	2.56	60.32
3	3.42	99.80	49.90	− 0.23	49.17	98.63	0.006	52.29	97.40	3.14	61.76
4	4.65	98.60	49.80	− 0.29	48.37	98.95	0.009	51.16	98.21	2.88	58.36

**Table 2:** Effect of Initial concentration on kinetic data thorium adsorption onto graphene oxide adsorbent functionalized with aminomethyl phosphinic acid (Experimental conditions: pH = 3, Adsorbent dosage = 2 g L^−1^, Time = 180 min, T = 298 K, Shaker speed = 200 rpm).

Initial concentration (mg L^−1^)	Adsorption (%)	q_exp_ (mg g^−1^)	Pseudo first-order			Pseudo second-order			Weber- Morris	
			k_1_	q_e (theoritical)_	R^2^	k_2_	q_e (theoritical)_	R^2^	k_i_	R^2^
50	99.90	24.97	− 0.34	24.05	98.59	0.020	25.42	99.77	1.399	57.96
100	99.80	49.90	− 0.23	49.17	98.63	0.006	52.29	97.40	3.1397	61.76
200	57.96	57.96	− 0.29	55.97	98.45	0.007	59.49	97.99	3.4605	61.47
500	43.86	109.65	− 0.19	108.48	98.76	0.002	116.66	96.54	7.5587	63.66

**Table 3:** Effect of Adsorbent dosage on kinetic data thorium adsorption onto graphene oxide adsorbent functionalized with aminomethyl phosphinic acid (Experimental conditions: Thorium concentration = 100 mg L^−1^, pH = 3, Time = 180 min, T = 298 K, Shaker speed = 200 rpm).

Adsorbent dosage (g L^−1^)	Adsorption (%)	q_exp_ (mg g^-1^)	Pseudo first-order			Pseudo second-order			Weber- Morris	
			k_1_	q_e (theoritical)_	R^2^	k_2_	q_e (theoritical)_	R^2^	k_i_	R^2^
0.1	12.19	121.90	− 0.21	120.52	98.27	0.0021	129.12	96.31	8.12	61.62
0.25	37.78	127.13	− 0.22	125.77	98.54	0.0022	134.45	96.93	8.29	60.77
0.5	65.93	131.86	− 0.23	130.30	98.67	0.0023	139.09	97.10	8.47	60.07
1	95.56	95.56	− 0.22	95.22	99.15	0.0029	101.74	96.74	6.28	60.12
2	99.80	49.90	− 0.23	49.17	98.63	0.006	52.29	97.40	3.14	61.76

**Table 4:** Effect of Temperature on kinetic data thorium adsorption onto graphene oxide adsorbent functionalized with aminomethyl phosphinic acid (Experimental conditions: Thorium concentration = 100 mg L^-1^, Adsorbent dosage = 0.5 g L^−1^, Time = 180 min, pH = 3, Shaker speed = 200 rpm).

Temperature (K)	Adsorption (%)	q_exp_ (mg g^−1^)	Pseudo first-order			Pseudo second-order			Weber- Morris	
			k_1_	q_e (theoritical)_	R^2^	k_2_	q_e (theoritical)_	R^2^	k_i_	R^2^
298	65.93	131.86	− 0.23	130.30	98.67	0.0023	139.08	97.10	8.47	60.07
308	67.01	134.02	− 0.24	132.65	98.71	0.0023	141.51	97.24	8.56	59.73
318	68.83	137.66	− 0.25	135.81	98.83	0.0023	144.82	97.34	8.70	59.40
328	69.42	138.84	− 0.25	136.54	99.04	0.0024	145.42	97.79	8.67	59.53

According to Table 2, the thorium adsorption capacity rose from 24.97 to 109.65 mg g^−1^ by varying the initial concentration of thorium solution within the range of 50 to 500 mg L^−1^.

Thorium adsorption was investigated using varying dosages of AMPA-GO (0.1, 0.25, 0.5, 1, and 2 g L^−1^). According to Table 3, the findings demonstrated that as AMPA-GO dosage is increased, the adsorption percentage rises due to an increase in adsorption sites. However, the ion adsorption capacity increased from 121.9 to 131.86 mg g^−1^ with increasing adsorbent dosage (0.1–0.5 g L^−1^) and then decreased with increasing adsorbent dosage.

The effect of temperature on adsorption was studied from 298 to 328 K. The results of Table 4 show that increasing the temperature of the solution increases thorium removal. Therefore, the adsorption of thorium by AMPA-GO is an endothermic process.

The results show that thorium adsorption by AMPA-GO adsorbent increases with increasing time. Less than 30 min are needed for the amount of adsorption to reach its maximum value; after that, the equilibrium condition is established and there is no discernible variation in the amount of adsorption. The values of the correlation coefficients (R^2^) indicate that the adsorption kinetics is more consistent with the pseudo-first-order model than the pseudo-second-order and Weber-Morris models. The Weber-Morris model's nonlinearity was demonstrated by the findings. Therefore, the adsorption process is not controlled by intraparticle diffusion ^49^ and thorium adsorption kinetics with this adsorbent is fast.

The minimum value of the average absolute error (AARE) for the experimental data was calculated with the following formula ^50^:

9 $$AARE= \frac{1}{n}\sum_{i=1}^{n}\left|\frac{{q}_{exp}-{q}_{pre.}}{{q}_{exp}}\right|,$$

where n represents the number of data points fitted into the models.

Table 5 displays the value of AARE for pseudo first-order and pseudo second-order models. The value of AARE for the pseudo first-order and pseudo second-order models data fitting was found to be in the range of 0.010–0.081 and 0.035–0.057, respectively. AARE values ​​show that the adsorption kinetics follow both models.

**Table 5:** The value of AARE for pseudo first-order and pseudo second-order models.

Parameters	AARE	
	Pseudo first-order	Pseudo second-order
pH	0.081	0.035
Adsorbent dosage	0.010	0.057
Initial concentration	0.024	0.039
Temperature	0.013	0.052

### Study of adsorption–desorption cycle

**Figure 6:**
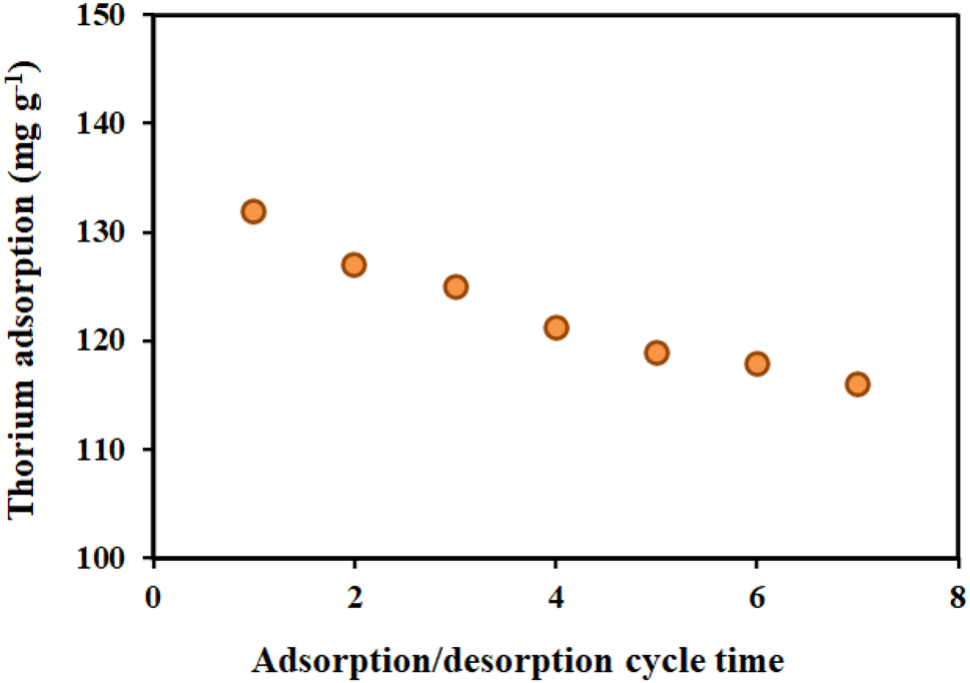
Changes in thorium adsorption by graphene oxide adsorbent functionalized with aminomethyl phosphinic acid in seven consecutive adsorption–desorption cycles (Experimental conditions: Thorium concentration = 100 mg L^−1^, Adsorbent dosage = 0.5 g L^−1^, Time = 180 min, T = 298 K, pH = 3, Shaker speed = 200 rpm).

From an economic perspective, adsorbent renewal plays a crucial role in adsorption. For this reason, seven cycles of thorium ion adsorption–desorption cycle from the AMPA-GO adsorbent were examined for 180 min at a temperature of 298 K, an initial thorium concentration of 100 mg L^-1^, pH equal 3, and an adsorbent dosage of 0.5 g L^−1^. Figure 6 displays the outcomes of the adsorption–desorption cycle. The same volume ratio, 0.5 M nitric acid and 0.1 M hydrochloric acid was used to desorb thorium ions from the adsorbents. Similar to the adsorption process, the desorption time of thorium cations from the adsorbent was calculated to be 180 min. After each desorption process, the adsorbents were separated by filter paper. After drying and weighing the adsorbent, the next adsorption test was performed. As seen in Fig. 2, the adsorption rate of thorium ions by the adsorbent has decreased from 131.86 mg g^-1^ in the first step to 116.06 mg g^-1^ in the seventh step. It can be said that the thorium adsorption capacity by the adsorbent has decreased by about 11% after seven adsorption–desorption cycles. Therefore, the results show that AMPA-GO adsorbent can be used seven times with an acceptable change in adsorption capacity. Research has demonstrated that one of the key elements supporting broader acceptability for their commercial application is adsorbent regeneration^51^. This ability of AMPA-GO adsorbent makes it competitive with other commercial adsorbents.

In addition to the ability to recover the adsorbent, the adsorption capacity is one of the main characteristics of an adsorbent. Table 4 shows that the thorium adsorption capacity with AMPA-GO adsorbent is 138.84 mg g^−1^. In Table 6, thorium adsorption by AMPA-GO adsorbent is compared with other adsorbents. The adsorption capacity of thorium by AMPA-GO adsorbent is higher than other adsorbents or is within the range of other adsorbents.

**Table 6:** Comparison of thorium adsorption by AMPA-GO adsorbent with other adsorbents.

Adsorbent	Adsorption capacity (mg g^-1^)	Reference	Adsorbent	Adsorption capacity (mg g^-1^)	Reference
Penicillium chrysogenum	142	^52^	Activated carbon	20.19	^53^
Amberlite XAD-4	58.01	^54^	Calcined diatomite	13.92	^55^
PAN/zeolite	9.28	^56^	Flux calcined diatomite	11.60	^55^
SiO_2_	0.23	^57^	Crystalline tin oxide nanoparticles	62.50	^58^
MX-80	63.81	^59^	Sodium bentonite	41.07	^60^
Amberlite XAD	26.22	^61^	Magnetic bentonite	31.33	^60^
Raw diatomite	6.96	^62^	Phosphate-grafted chitin (PGC)	50.5	^63^
Modified clay MTTZ	26.92	^64^	SA/PVA/PEO/ZSM5 nanohybrid adsorbent	139.2	^6^
Perlite	5.80	^22^	AMPA-GO	138.84	This work
Attapulgite	15.55	^65^			

### Effect of the co-existing ions

**Figure 7:**
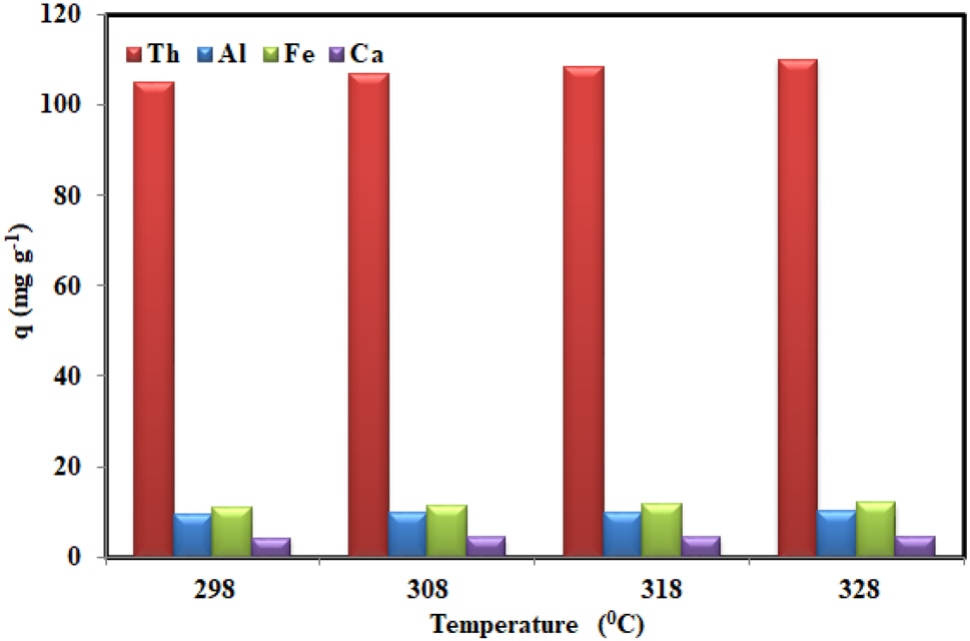
The comparison of the adsorption of Th, Al, Fe, and Ca at different temperatures (Experimental conditions: Thorium concentration = 98 mg L^−1^, Al concentration = 34.5 mg L^−1^, Fe concentration = 51 mg L^−1^, Ca concentration = 12.6 mg L^−1^, Adsorbent dosage = 0.5 g L^−1^, Time = 180 min, Shaker speed = 200 rpm).

The co-existing (Al, Fe, and Ca) ions effect on the performance of the AMPA-GO adsorbent in real system was investigated. Figure 7 shows a comparison of the adsorption of thorium, aluminum, calcium, and iron at an adsorbent dosage of 0.5 g L^−1^, and a temperature of 298–328 K. It can be seen that the adsorption capacity for all elements increased with the increase in temperature. The results show that AMPA-GO adsorbent always has a greater tendency to absorb thorium than other elements. Therefore, the AMPA-GO adsorbent can be used as a thorium adsorbent from real wastewaters.

### Continuous adsorption in a fixed bed column

#### Effect of the feed flow rate

**Figure 8:**
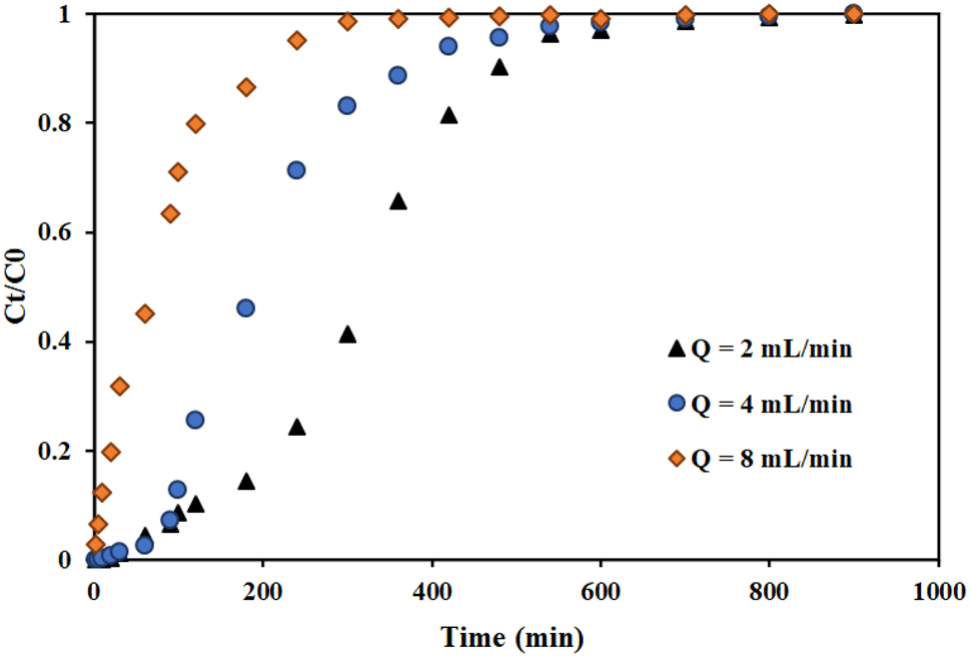
Effect of the flow rate in the fixed bed column containing graphene oxide adsorbent functionalized with aminomethyl phosphinic acid (Experimental conditions: thorium concentration = 100 mg L^−1^, bed height = 4 cm).

The results of the experiments to investigate the effect of flow rate in the values of 2, 4, and 8 mL min^−1^ are shown in Fig. 8. The bed height and thorium concentration in the inlet stream were 4 cm and 100 mg L^−1^, respectively. As Fig. 8 shows, the breakthrough curve happened faster as the input feed flow rate increased. The slope of the curves increased by increasing the flow rate from 2 to 8 mL min^−1^. The findings show that with increasing flow rate, the residence time of the solution in the column decreases. As a result, adsorption capacity of thorium in the column have decreased as the flow rate has increased ^66,67^. These results were consistent with other reported findings ^67^.

#### Effect of the column bed height

**Figure 9:**
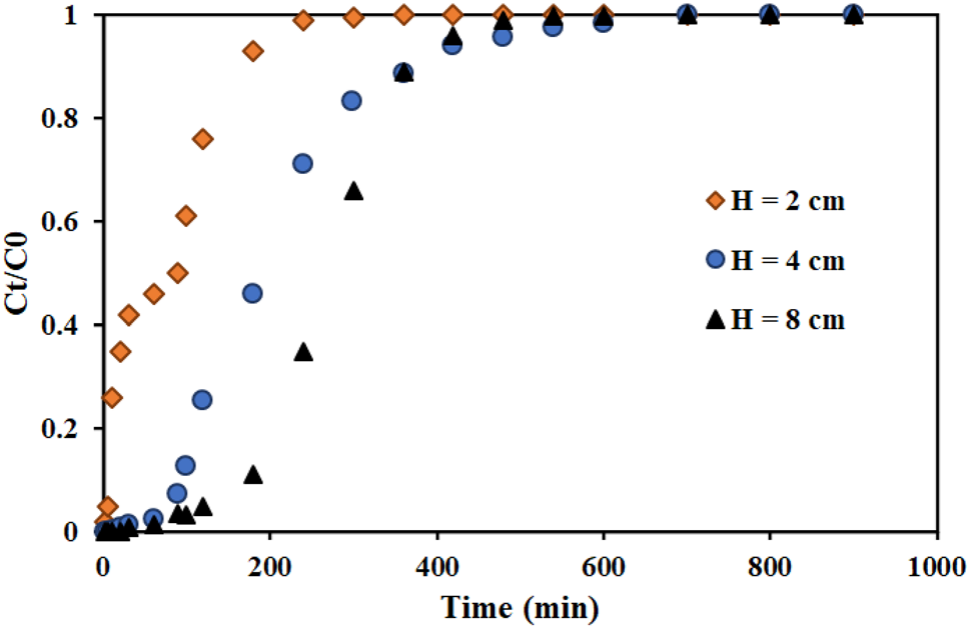
Effect of the column bed height of the graphene oxide adsorbent functionalized with aminomethyl phosphinic acid in the fixed bed column (Experimental conditions: thorium concentration = 100 mg L^−1^, flow rate = 4 mL min^−1^).

Figure 9 shows the findings of evaluating the influence of bed height containing the functionalized graphene oxide adsorbent in amounts of 2, 4, and 8 cm on thorium ion adsorption. The breakthrough curve's slope drops while the column's adsorption capacity, breakthrough time, and saturation time rise with increasing bed height (more adsorbent mass). The batch studies showed that a time 30 min for thorium adsorption with AMPA-GO adsorbent is necessary. Therefore, the low height of the column bed, the AMPA-GO adsorbent does not find enough time to completely absorb the ions. This phenomenon is observed at H equal to 2 cm. Therefore, for the optimal efficiency of the fixed bed column, a minimum height of the column bed is always necessary. So, a height of the column bed of 4 cm was chosen.

#### Effect of the feed concentration

**Figure 10:**
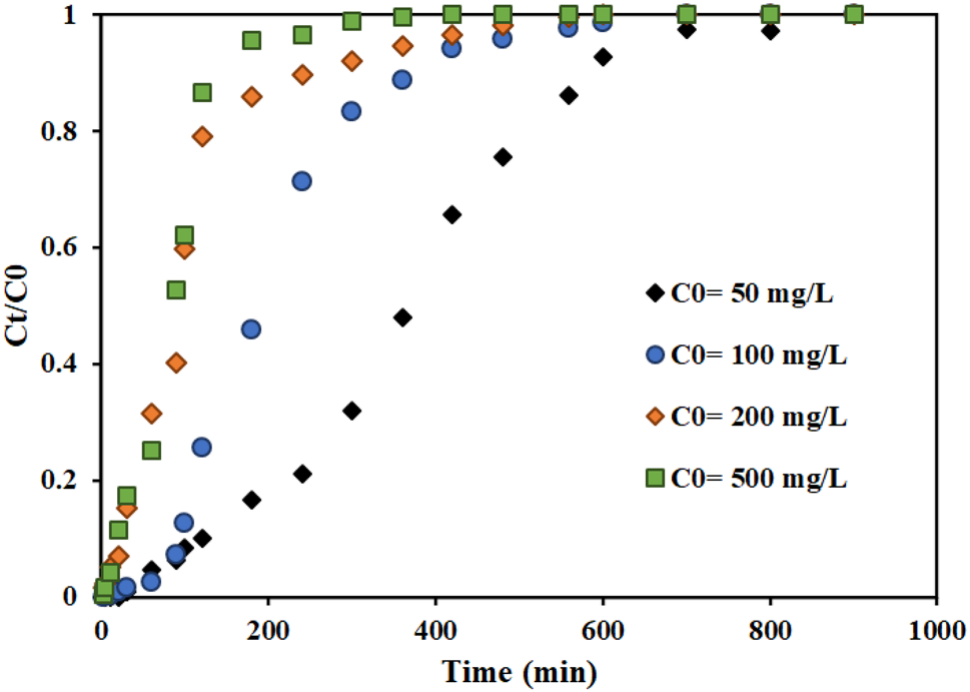
Effect of the feed concentration on the performance of fixed bed column containing the graphene oxide adsorbent functionalized with aminomethyl phosphinic acid (Experimental conditions: flow rate = 4 mL min^−1^, bed height = 4 cm).

With the presence of solutions with different concentrations, it is necessary to investigate the effect of the input feed concentration on the performance and adsorption capacity of the fixed bed column. In the previous research, the effects of different initial concentrations of thorium were investigated in batch systems with AMPA-GO adsorbent. The findings demonstrated that the highest amount of adsorption was 178.67 mg g^-1^ and increased the adsorption capacity with increasing concentration ^40^. The current research investigated the effect of thorium ion concentration in the range of 50 to 500 mg L^-1^ on the thorium ion adsorption using functionalized graphene oxide adsorbent in a fixed bed column. Figure 10 displays the breakthrough curves for various thorium concentrations in the input feed to the fixed bed column. The findings demonstrated that the breakthrough and saturation times are accelerated by increasing the thorium concentration in the feed stream.

As the initial thorium concentration increased from 50 to 500 mg L^-1^, the progress curves became steeper, and due to less mass transfer from the bulk solution to the surface of AMPA-GO, the breakthrough volume decreased ^68^.

#### Investigation of kinetic models in fixed bed column

**Figure 11:**
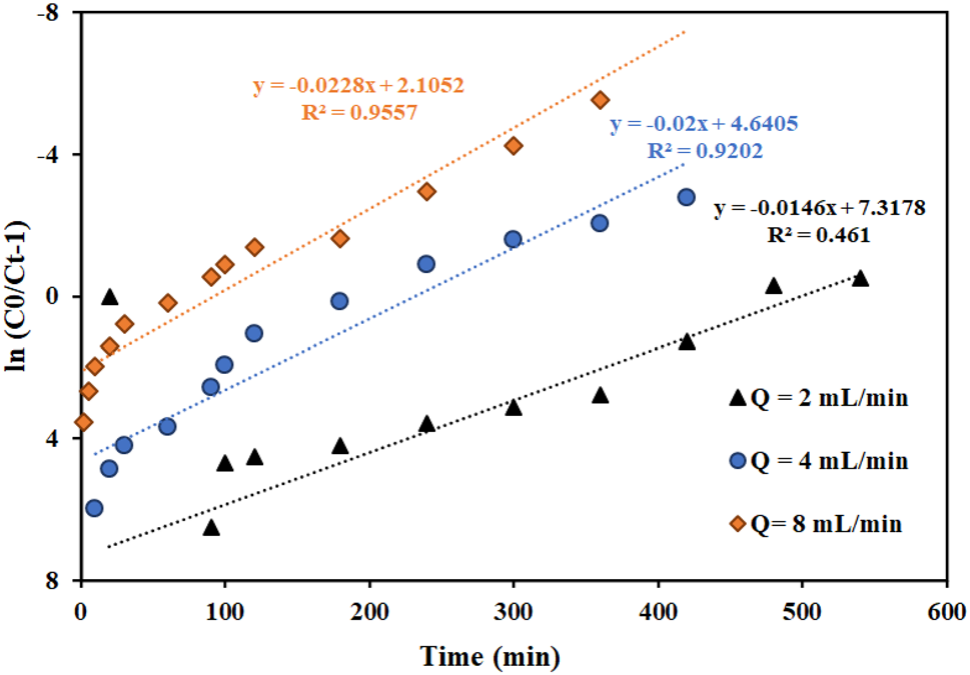
Linear plot of the Thomas model with experimental data at different flow rates (Experimental conditions: flow rate = 4 mL min^−1^, bed height = 4 cm).

**Figure 12:**
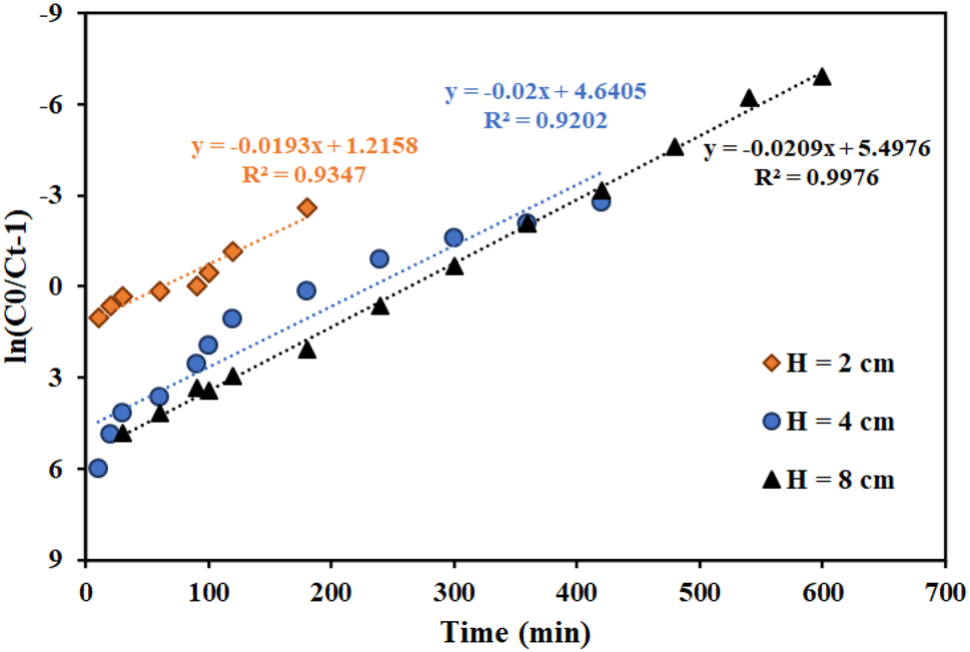
Linear plot of the Thomas model with experimental data at different column bed heights (Experimental conditions: flow rate = 4 mL min^−1^, bed height = 4 cm).

**Figure 13:**
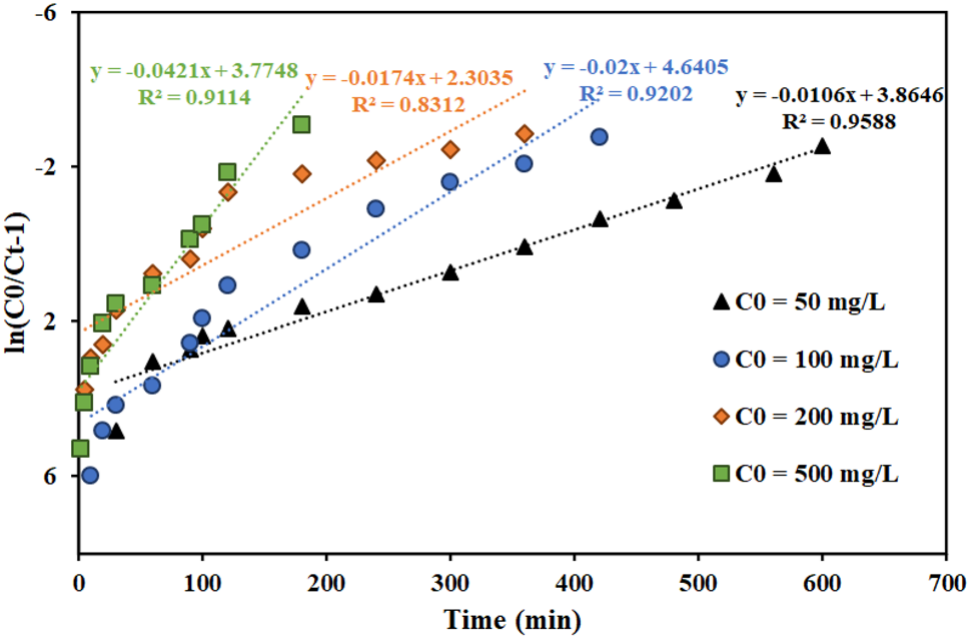
Linear plot of the Thomas model with experimental data at different feed concentrations (Experimental conditions: flow rate = 4 mL min^−1^, bed height = 4 cm).

Figures 11, 12, 13 and Table 7 present the Th adsorption with the Thomas model. The outcomes demonstrated that the Thomas model adequately describes the experimental data and that the correlation coefficient (R^2^) is within a reasonable range. As the flow rate increased, the Thomas rate constant increased from 0.140 to 0.228 L mg^−1^ min^−1^. The value of q_0_ rises in proportion to the desired component's concentration. As a result, the adsorption process is carried out with a greater driving force because of the difference between the concentration of thorium ions adsorbed on the graphene oxide adsorbent and its concentration in the solution. The constant values of the Thomas velocity decrease with increasing height of the graphene oxide adsorbent bed. In this case, the breakdown time and column discharge time increase as the adsorbent bed height does, as would be predicted.

**Table 7:** Thomas model parameters for thorium adsorption in different conditions of fixed bed column containing graphene oxide adsorbent functionalized with aminomethyl phosphinic acid (Experimental conditions: flow rate = 4 mL min^−1^, bed height = 4 cm).

Concentration (mg L^−1^)	Flow rate (mL min^−1^)	Bed height (cm)	K_Th_ × 10^3^ (L mg^−1^ min^−1^)	q_0_ (mg g^−1^)	R^2^
100	2	4	0.140	23.942	0.9557
100	4	4	0.200	21.278	0.9202
100	8	4	0.228	16.935	0.8364
100	4	2	0.193	11.377	0.9371
100	4	8	0.209	12.235	0.9812
50	4	4	0.212	16.717	0.9588
200	4	4	0.087	24.281	0.8312
500	4	4	0.084	41.113	0.9114

**Figure 14:**
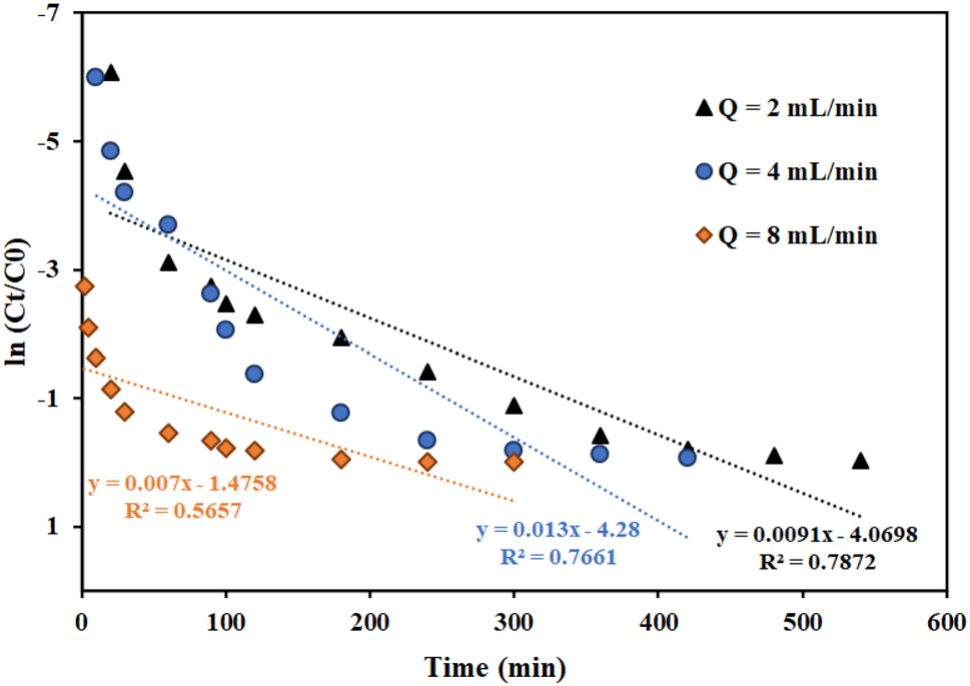
Linear plot of the Bohart–Adams model with experimental data at different flow rates (Experimental conditions: flow rate = 4 mL min^−1^, bed height = 4 cm).

**Figure 15:**
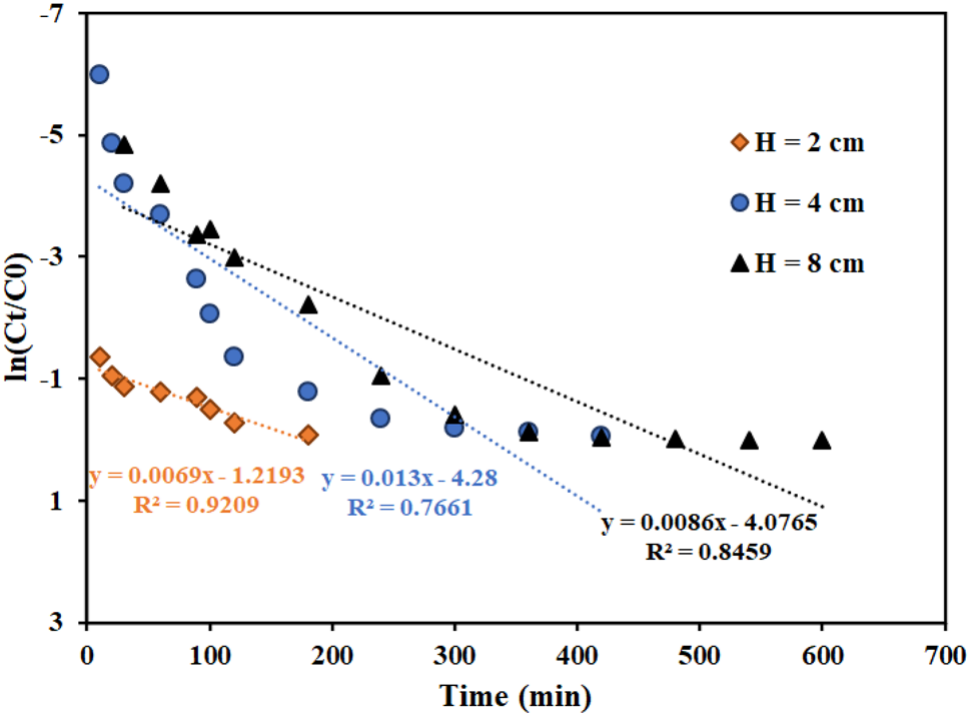
Linear plot of the Bohart–Adams model with experimental data at different column bed heights (Experimental conditions: flow rate = 4 mL min^−1^, bed height = 4 cm).

**Figure 16:**
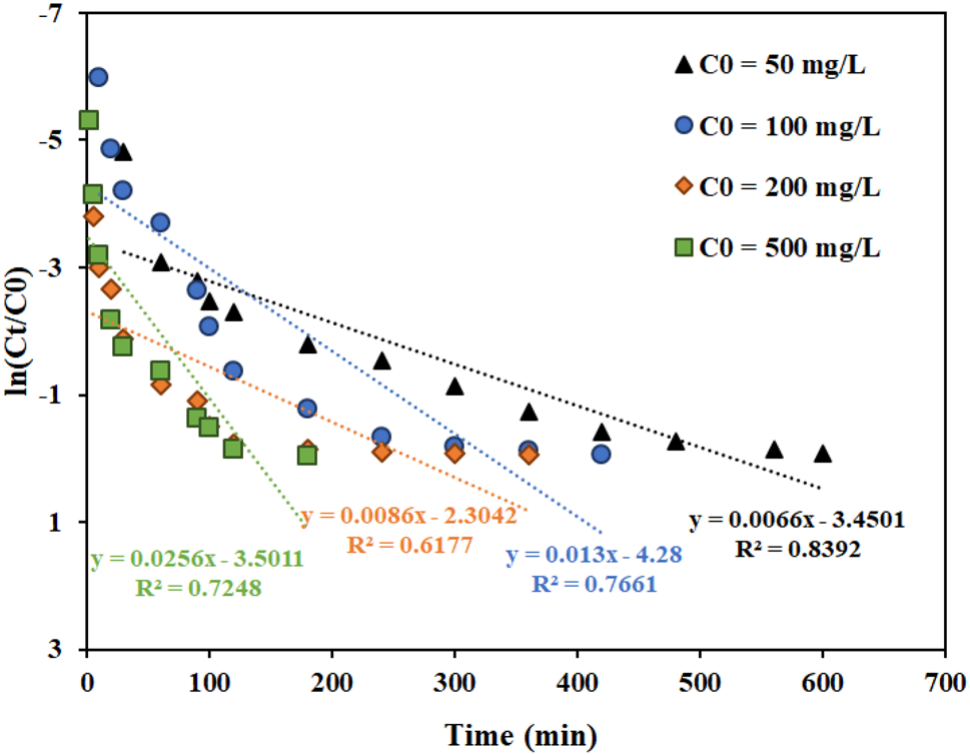
Linear plot of the Bohart–Adams model with experimental data at different feed concentrations (Experimental conditions: flow rate = 4 mL min^−1^, bed height = 4 cm).

According to the results of fitting the Adams-Bohart model in Table 8 and Figs. 14, 15, 16, with flow rate increase from 2 to 8 mL min^−1^, the maximum volume adsorption capacity (N_0_) has increased from 19,788 to 37,314 mg L^−1^. The results showed that the initial ion concentration had a similar effect. Meanwhile, with the increase in flow rate and initial concentration of ions, the rate constant (K_AB_) has decreased.

**Table 8:** Bohart–Adams model parameters for thorium adsorption in different conditions of fixed bed column containing graphene oxide adsorbent functionalized with aminomethyl phosphinic acid (Experimental conditions: flow rate = 4 mL min^-1^, bed height = 4 cm).

Concentration (mg L^−1^)	Flow rate (mL min^−1^)	Bed height (cm)	K_AB_ × 10^4^ (L mg^−1^ min^−1^)	N_0_ (mg L^−1^)	R^2^
100	2	4	0.91	19,788.97	0.7872
100	4	4	1.30	29,135.47	0.7661
100	8	4	0.70	37,314.79	0.6176
100	4	2	0.69	28,967.55	0.8305
100	4	8	0.86	20,973.97	0.8948
50	4	4	1.32	23,130.20	0.8392
200	4	4	0.43	47,421.28	0.6177
500	4	4	0.512	60,514.03	0.7248

**Figure 17:**
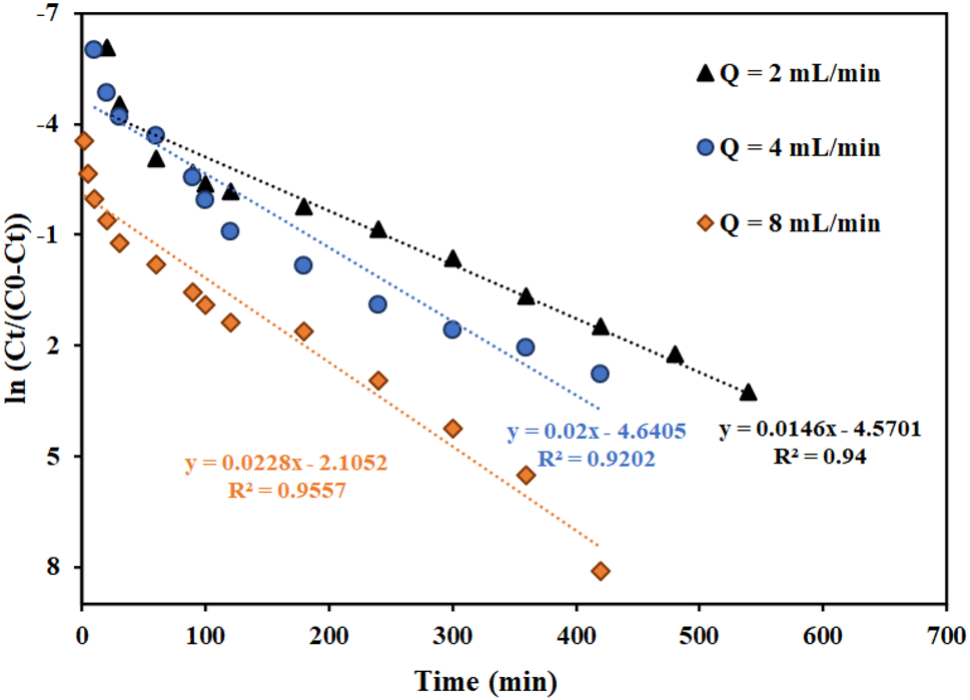
Linear plot of the Yoon-Nelson model with experimental data at different flow rates (Experimental conditions: flow rate = 4 mL min^−1^, bed height = 4 cm).

**Figure 18:**
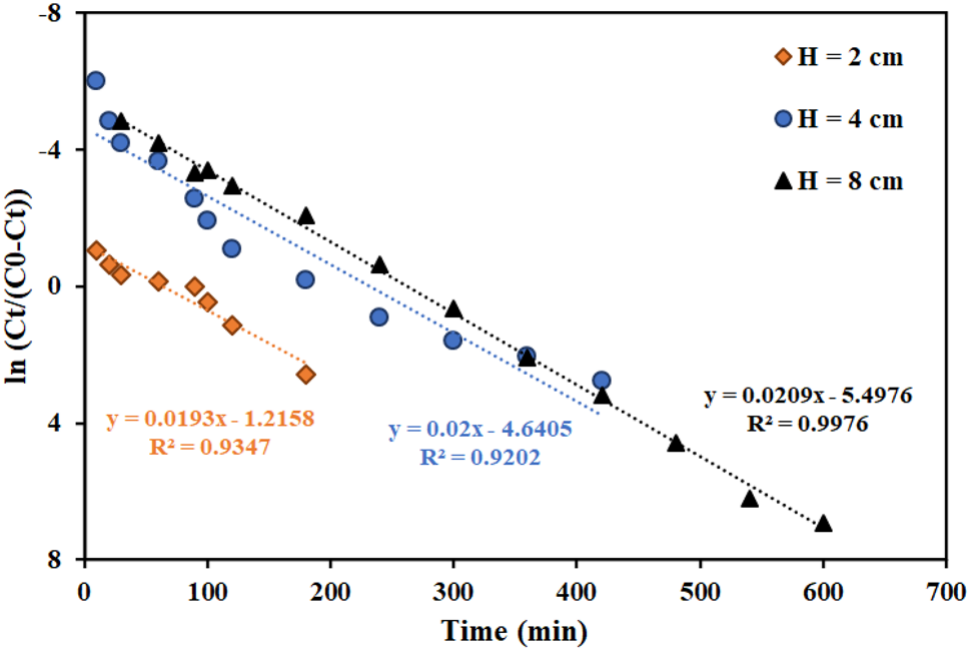
Linear plot of the Yoon-Nelson model with experimental data at different column bed heights (Experimental conditions: flow rate = 4 mL min^−1^, bed height = 4 cm).

**Figure 19:**
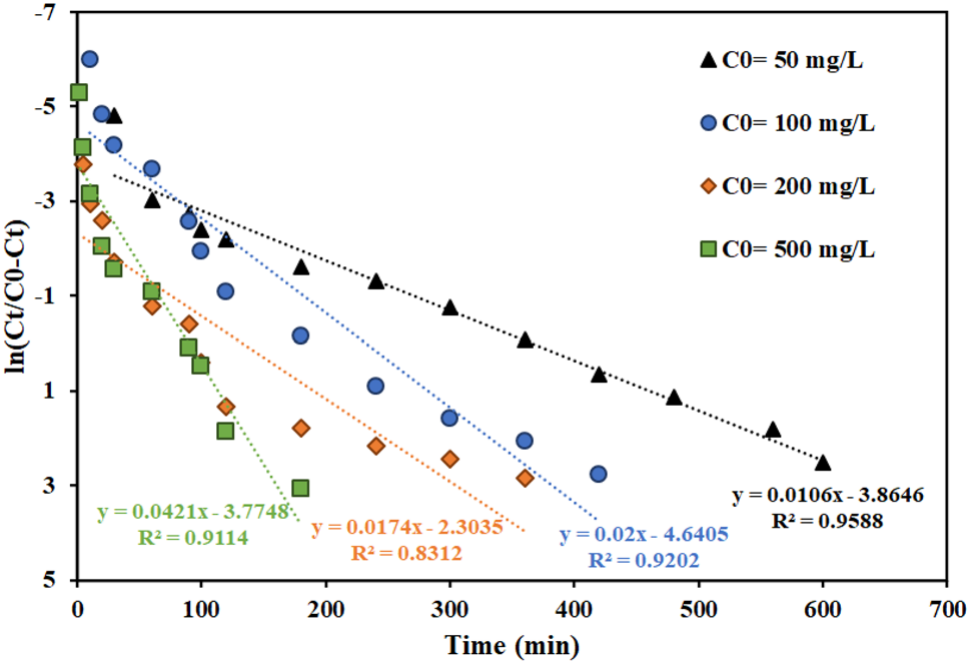
Linear plot of the Yoon-Nelson model with experimental data at different feed concentrations (Experimental conditions: flow rate = 4 mL min^−1^, bed height = 4 cm).

Figures 17, 18 and 19 and Table 9 display the fitting results using the Yoon-Nelson model. The outcomes demonstrate that both the fit with the Thomas model and the correlation coefficient (R^2^) values with the Yoon-Nelson model are in good agreement with the laboratory data. This model states that τ and the K_YN_ increases as the flow rate increases from 2 to 8 min mL^-1^. Additionally, by raising the bed's height from 2 to 8 cm, τ has increased from 62 to 263 min because of the increase in adsorbent's amount and contact area with thorium ions. The value of τ dropped as the initial thorium concentration was increased from 50 to 500 mg L^−1^.

**Table 9:** Yoon-Nelson model parameters for thorium adsorption in different conditions of fixed bed column containing graphene oxide adsorbent functionalized with aminomethyl phosphinic acid (Experimental conditions: flow rate = 4 mL min^−1^, bed height = 4 cm).

Concentration (mg L^−1^)	Flow rate (mL min^−1^)	Bed height (cm)	K_YN_ (min^−1^)	τ (min)	R^2^
100	2	4	0.0146	313.02	0.9400
100	4	4	0.0200	232.02	0.9202
100	8	4	0.0228	92.33	0.9557
100	4	2	0.0193	62.99	0.9317
100	4	8	0.209	263.04	0.9976
50	4	4	0.106	364.585	0.9958
200	4	4	0.0174	132.385	0.8312
500	4	4	0.0421	89.663	0.9114

According to the findings of the linear graphs of the three models (the Bohart-Adams, Yoon-Nelson, and Thomas), the fixed bed system can be effectively described by the Thomas and Yoon-Nelson models. However, the Bohart-Adams model is unsuitable for predicting the laboratory data of thorium adsorption with AMPA-GO adsorbent due to the low correlation coefficient (R^2^ less than 0.9).

## Conclusions

In this research, graphene oxide was synthesized and functionalized using aminomethylphosphonic acid (AMPA). The adsorbent capability of functionalized graphene oxide was studied for thorium adsorption from an aqueous solution. First, the AMPA-GO adsorbent was characterized using TEM, XRD, and FTIR methods. Thorium adsorption experiments were performed in batch and continuous modes, and the adsorption kinetic studies were carried out accurately.

In batch experiments, as the pH increased from 1 to 3, the adsorption capacity increased from 29.25 to 49.90 mg g^−1^. Thorium adsorption capacity rose from 24.97 to 109.65 mg g^−1^ by varying the initial concentration of thorium solution within the range of 50 to 500 mg L^−1^. The thorium adsorption capacity increased from 121.9 to 131.86 mg g^−1^ with increasing adsorbent dosage (0.1–0.5 g L^−1^) and then decreased from 131.86 to 49.9 mg g^−1^ with increasing adsorbent dosage (0.5–2 g L^−1^). Also, the effect of temperature on adsorption was studied from 298 to 328 K. The temperature study showed that the thorium adsorption by AMPA-GO is an endothermic process.

The maximum experimental adsorption capacity of thorium ions was observed 138.84 mg g^−1^ at a pH of 3, adsorbent dosage of 0.5 g L^−1^, and a temperature of 328 K. The results of thorium adsorption in batch mode were investigated with three pseudo-first-order, pseudo-second-order, and Weber- Morris kinetic models. The data were more consistent with the pseudo-first-order model. The results of the experiments showed that the AMPA-GO adsorbent has a good ability to absorb thorium up to seven adsorption–desorption cycles.

The effect of feed flow rate (2–8 mL min^−1^), initial concentration (50–500 mg L^-1^), and column bed height (2–8 cm) on the adsorption of thorium ions in the fixed bed column was scrutinized. With increasing flow rate from 2 to 8 mL min^−1^ and initial concentration from 50 to 500 mg L^−1^, the breakthrough curve happened faster. While increasing the column bed height up to 8 cm, the column showed good adsorption capacity for a long time. The fixed bed column's performance was assessed using the kinetic models of Thomas, Yoon-Nelson, and Bohart-Adams. Under identical experimental conditions, the Bohart-Adams model's correlation coefficients were frequently lower than those of the Thomas and Yoon-Nelson models.

## Data Availability

The datasets used and/or analyzed during the current study are available from the corresponding author on reasonable request.

## Abbreviations

*AMPA*: Aminomethyl phosphinic acid
*GO*: Graphene oxide
*C*_*0*_: Initial adsorbate concentration (mg L^−^^1^)
*C*_*e*_: Equilibrium adsorbate concentration (mg L^−^^1^)
*C*_*t*_: Adsorbate concentration at time *t* (mg L^−^^1^)
N_0_: Saturation concentration (mg L^−^^1^)
Z: Column bed height (cm)
K_AB_: Bohart-Adams rate constant (L mg^−^^1^ min^−^^1^)
U_0_: Linear flow rate (cm min^−^^1^)
Q: Flow rate (mL min^−^^1^)
K_Th_: Thomas rate constant (L mg^−^^1^ min^−^^1^)
M: Mass of adsorbent (g)
τ: The time required for 50% adsorbate breakthrough (min)
K_YN_: Yoon-Nelson constant (min^−^^1^)
*V*: Solution volume (mL)
*k*_1_: Pseudo-first-order rate constant (min^−^^1^)
*k*_2_: Pseudo-second-order rate constant (g mg^−^^1^ min^−^^1^)
*k*_*i*_: Weber- Morris model constant (mg g^−^^1^ min^−^^0.5^)
*q*_*e*_: Adsorption capacity at equilibrium (mg g^−^^1^)
*q*_*t*_: Adsorption capacity at time *t* (mg g^−^^1^)
*R*^*2*^: Determination coefficient
*t*: Time (min)
*T*: Temperature (K)
